# Spatial Distribution and Conservation of Speckled Hind and Warsaw Grouper in the Atlantic Ocean off the Southeastern U.S.

**DOI:** 10.1371/journal.pone.0078682

**Published:** 2013-11-19

**Authors:** Nicholas A. Farmer, Mandy Karnauskas

**Affiliations:** 1 National Oceanographic and Atmospheric Administration, National Marine Fisheries Service, Southeast Regional Office, St. Petersburg, Florida, United States of America; 2 National Oceanographic and Atmospheric Administration, National Marine Fisheries Service, Southeast Fisheries Science Center, Miami, Florida, United States of America; North Carolina State University, United States of America

## Abstract

There is broad interest in the development of efficient marine protected areas (MPAs) to reduce bycatch and end overfishing of speckled hind (*Epinephelus drummondhayi*) and warsaw grouper (*Hyporthodus nigritus*) in the Atlantic Ocean off the southeastern U.S. We assimilated decades of data from many fishery-dependent, fishery-independent, and anecdotal sources to describe the spatial distribution of these data limited stocks. A spatial classification model was developed to categorize depth-grids based on the distribution of speckled hind and warsaw grouper point observations and identified benthic habitats. Logistic regression analysis was used to develop a quantitative model to predict the spatial distribution of speckled hind and warsaw grouper as a function of depth, latitude, and habitat. Models, controlling for sampling gear effects, were selected based on AIC and 10-fold cross validation. The best-fitting model for warsaw grouper included latitude and depth to explain 10.8% of the variability in probability of detection, with a false prediction rate of 28–33%. The best-fitting model for speckled hind, per cross-validation, included latitude and depth to explain 36.8% of the variability in probability of detection, with a false prediction rate of 25–27%. The best-fitting speckled hind model, per AIC, also included habitat, but had false prediction rates up to 36%. Speckled hind and warsaw grouper habitats followed a shelf-edge hardbottom ridge from North Carolina to southeast Florida, with speckled hind more common to the north and warsaw grouper more common to the south. The proportion of habitat classifications and model-estimated stock contained within established and proposed MPAs was computed. Existing MPAs covered 10% of probable shelf-edge habitats for speckled hind and warsaw grouper, protecting 3–8% of speckled hind and 8% of warsaw grouper stocks. Proposed MPAs could add 24% more probable shelf-edge habitat, and protect an additional 14–29% of speckled hind and 20% of warsaw grouper stocks.

## Introduction

The South Atlantic Fishery Management Council (SAFMC) manages speckled hind (*Epinephelus drummondhayi*) and warsaw grouper (*Hyporthodus nigritus*) from federal waters at the Virginia/North Carolina border through the Atlantic side of the Florida Keys. Currently, these stocks are listed as undergoing overfishing, with an unknown overfished status [1]. Stock assessments of varying degrees of resolution and rigor have indicated a declining trend for both stocks [2]–[10]. In the first formal stock assessment of speckled hind and warsaw grouper [11], catch curve analyses indicated that static spawning potential ratios (SPR) for warsaw grouper were between 0.2% and 6% in 1988 and 1990, and speckled hind SPR values declined from 25% in 1988 to 5% in 1999 [4]–[7]. SPR is the average fecundity of a recruit over its lifetime when the stock is fished divided by the average fecundity of a recruit over its lifetime when the stock is unfished; the low ratios from the most recent assessment [11] indicated the stocks were undergoing overfishing. A recent study [10] sampled 1,365 speckled hind (1977–2007) from North Carolina to central Florida and revealed trends suggesting speckled hind are overfished and undergoing overfishing, including increasing fishing mortality rate, decreasing size-at-age, and reduced numbers of mature individuals. There is a broad scientific and management interest in the development of effective and efficient regulations to reduce bycatch mortality and promote the rebuilding for these stocks.

Speckled hind and warsaw grouper have a complicated management history which makes any analysis of their distribution or current status from fishery-dependent data analytically challenging. Speckled hind and warsaw grouper regulations went from inclusion in the five grouper aggregate recreational bag limit in 1992 (56 FR 56016), to a commercial and recreational limit of one per vessel of each species with a commercial sale prohibition of these species in 1994 (59 FR 27242), to a complete harvest prohibition of both species in 2011 (75 FR 82280). In February 2009, Amendment 14 to the SAFMC's Snapper-Grouper Fishery Management Plan (S-G FMP) implemented eight deepwater marine protected areas (MPAs), in part to reduce bycatch of speckled hind and warsaw grouper. Due to continuing concerns regarding the status of these stocks, Amendment 17B established annual catch limits (ACLs) of zero pounds for speckled hind and warsaw grouper in January 2011and prohibited harvest beyond a depth of 240 ft (73.15 m) for snowy grouper, blueline tilefish, yellowedge grouper, misty grouper, queen snapper, and silk snapper in the U.S. South Atlantic. In May 2012, Regulatory Amendment 11 (Reg-11) to the S-G FMP removed the 240-ft closure to deep-water species imposed by Amendment 17B, in favor of more targeted, shelf-edge spatial protection.

**Figure 1:**
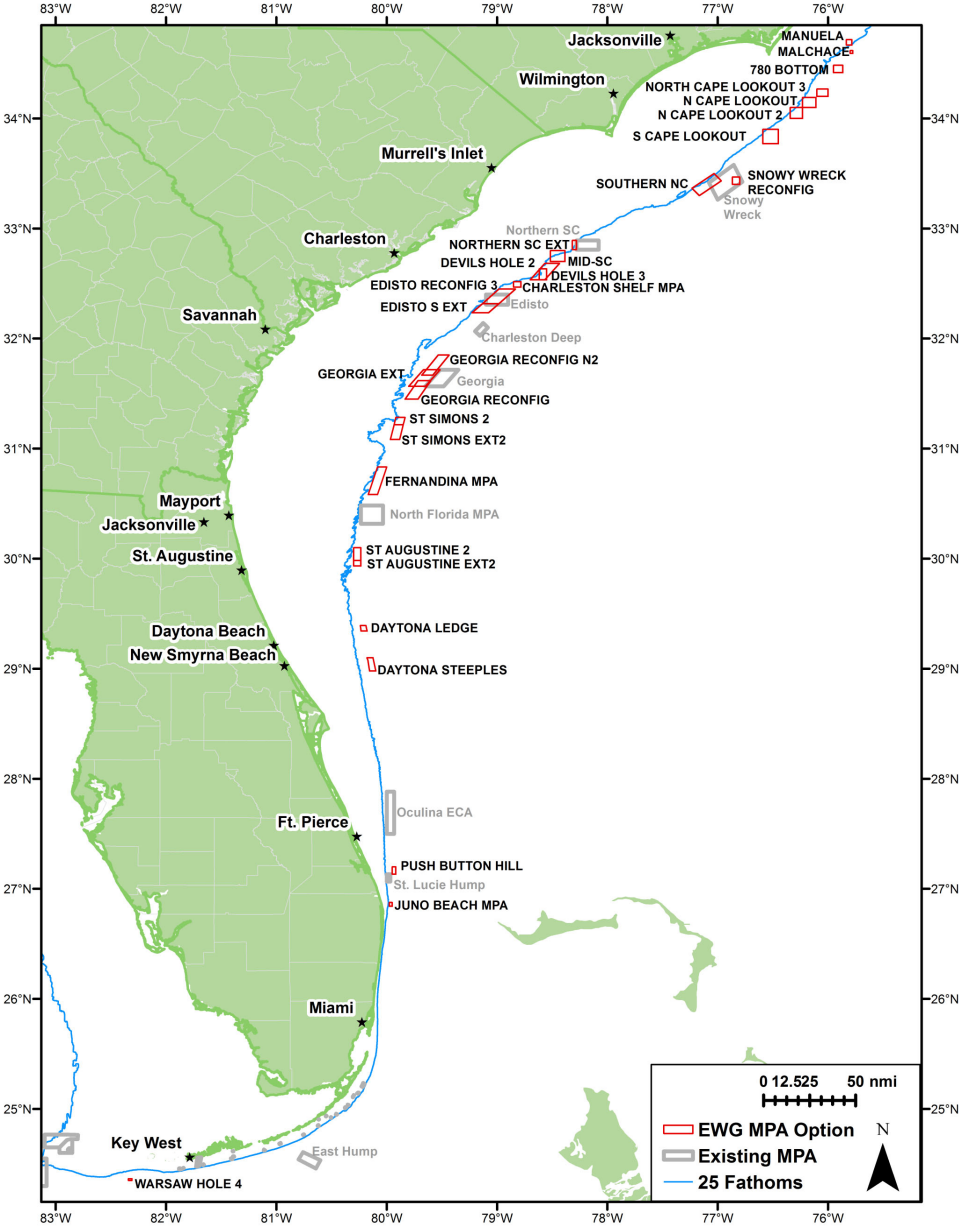
Existing and proposed protected areas. Existing (gray) marine protected areas (MPAs) and MPA options (red) developed by the South Atlantic Fisheries Management Council's MPA Expert Workgroup (SAFMC 2013).

To provide greater protection to these species, the SAFMC is currently developing Regulatory Amendment 17 (Reg-17), which proposes a variety of spatial closures which could reduce bycatch mortality for these stocks. A broad suite of no-take marine protected area (MPA) alternatives were developed by the SAFMC MPA Expert Working Group (EWG; Fig. 1). The analysis presented in this paper assimilates all available fishery-dependent and fishery-independent data to describe the geographic distribution of speckled hind and warsaw grouper. The relative conservation benefits of existing and proposed MPAs are also evaluated for each stock.

## Methods

### Data Sources

To determine locations of warsaw grouper and speckled hind encounters, observations were compiled from numerous sources (Table 1) and merged into a Geographic Information System (GIS) database. All data were used to develop a spatial classification model, and select data with underlying effort information were used to develop a more rigorous geographic distribution model. Locations of catch were provided to the highest possible resolution. Each of the data sets is described in detail below.

**Table 1:** Point data sources evaluated in meta-analysis, indicating whether data was included in spatial classification model (SCM) or geographic distribution model (GDM).

Data Source	Years	Resolution	Discards?	Depth?	SCM	GDM
Headboat Log	1973–2011	Some 0.17°×0.17°	2004-present	No	X	X
Reef Fish Observer	2006–2011	Lat/Long	Yes	Yes	X	X
MARMAP	1977–2011	Lat/Long	Yes	Yes	X	X
DW ROV Survey	2004–2011	Lat/Long	Yes	Not provided	X	X
Fisher Reports	1960s–2011	Loran and Lat/Long	Yes	Some	X	
SEFIS	2010–2011	Lat/Long	Yes	Yes	X	X
REEF	1980s–2011	Lat/Long	Yes	No	X	
Oculina ROV	2003–2005	Lat/Long	Yes	Yes	X	X
Manooch Fisheries Research Group (FRG)	1972–1977	Lat/Long	Yes	Yes	X	X
Sedberry Sub	1985, 2002	Lat/Long	Yes	Yes	X	
Rudershausen et al.	2007	Lat/Long	Yes	Yes	X	
Museum Collections	1884–1991	Lat/Long	Yes	Yes	X	

Since 1977, the Marine Resources Monitoring Assessment and Prediction (MARMAP) program has conducted fisheries-independent research between Cape Lookout, North Carolina, and Ft. Pierce, Florida. Gears and methodologies used have been consistent over the years to allow for long term analysis and comparisons. Sampling effort for snapper-grouper has historically been concentrated off South Carolina using various trap gears. MARMAP samples accurately identify fish to species and also collect valuable information on undersized fish. MARMAP data were aggregated by gear and set (i.e., a single trap, or a single line).

Since 2010, National Marine Fisheries Service's Southeast Fisheries Science Center (NMFS-SEFSC) has conducted fishery independent Southeast Fishery-Independent Survey video (SEFIS-V) and trap surveys (SEFIS-T). All gear-set level point observations of speckled hind and warsaw grouper from these surveys were incorporated into GIS. Additional survey data from submersible dives on continental shelf edge habitats were also incorporated (http://www.sefsc.noaa.gov/labs/beaufort/ecosystems/sefis/).

Since 2004, NMFS has conducted deep-water remote-operated vehicle (DW-ROV) surveys of the five natural bottom MPAs in the US South Atlantic between Jacksonville, FL and Cape Fear, NC. Based upon limited multibeam bathymetric maps and the local knowledge of other researchers, ROV transects were surveyed inside the MPAs and in adjacent open-to-fishing areas of similar depth and habitat type. Transects of roughly 1 km were followed and the resulting videotapes were analyzed for all detected fish and structure forming invertebrates. Observations of speckled hind and warsaw grouper were identified in a GIS map. Additional information using similar methods were collected in the Oculina Banks MPA from 2001–2005 (Oculina-ROV).

Since 1973, the Southeast Headboat Survey (HBS) has required recreational headboat captains to maintain logbooks recording trip-level information on number of anglers, trip duration, date, area fished, and catch by species. Headboats typically accommodate 15 or more anglers on half- or full-day for-hire trips. Headboat encounters (landings plus releases) were summarized by species, year, month, and area fished. Reporting of area fished has improved through time, with resolution ranging from state level to 0.17° by 0.17° grids. Area fished is self-reported, and vessels fishing in multiple areas during a trip were constrained by the data form design to report only one area fished for the trip. As such, the spatial reliability of headboat data, especially for rarely encountered species, is questionable. Depth fished was not reported.

In July 2006, NMFS-SEFSC began a voluntary reef fish observer program (RFOP) to characterize fishery landings and bycatch in the Atlantic Ocean off the southeastern U.S. This program is limited in geographic scope, but provides accurate set-level geographic location and discard information for fish encountered using bottom longline, electric (bandit) reel, and hand lines. Depth fished was reported for each set.

Between 1972–1979, scientists from NMFS-SEFSC's Fisheries Research Group (FRG) collected fish from offshore waters between Cape Lookout and Cape Fear, North Carolina. Numbers, size, and collection location were recorded by species at three primary sites in Onslow Bay, NC [8].

Since 1990, the Reef Environmental Education Foundation (REEF) survey has collected standardized information from volunteer divers and snorkelers on marine fish populations. Using a roving diver technique, volunteers recorded the geographic location and approximate abundance of species sited (www.reef.org). Only two REEF speckled hind records, one by a novice and one by an expert, were incorporated into the analysis; both observed two speckled hind on the USS Wilkes Barre wreck.

From 1979 to 2012, NMFS-SEFSC and the University of Miami, in conjunction with various federal, state and academic partners, have conducted a reef fish visual census (RVC) in the Florida Keys and Dry Tortugas [12]. In this two-stage sampling design, trained divers conduct a stationary point count of all reef fish stocks within a given distance of the sampling site, and record species, abundance, and various size metrics.

In 1985 and 2002, Dr. George Sedberry (South Carolina Department of Natural Resources) participated in research submarine dives off the southeastern U.S. (Sedberry-Sub). Speckled hind and warsaw grouper were observed during some of these dives and the locations of the observations were recorded.

Since 1884, various U.S. museums have maintained collections of speckled hind and warsaw grouper, including the Florida Museum of Natural History, Gainesville (www.flmnh.ufl.edu/scripts/dbs/fish_pub.asp), the North Carolina State Museum of Natural Sciences, Raleigh (www.naturalsciences.org), and Smithsonian National Museum of Natural History, Washington, DC (www.mnh.si.edu). Geographic coordinates for capture locations were either downloaded directly from online catalogs or specifically requested (W. Laney, United States Fish and Wildlife Service, pers. comm.).

Through public comment and a series of expert workshops, several recreational and commercial fishermen contributed catch location information to SAFMC staff. An additional warsaw grouper site was identified from two complementary sources [13], [14]. Historical photographs and underwater videos were used to groundtruth several anecdotal sites.

### Spatial classification model

To classify shelf-edge habitats in the Atlantic Ocean off the southeastern U.S. with regards to their utility to speckled hind and warsaw grouper, a simple spatial classification model was developed as follows. Offshore habitats between 25–100 fathoms (45.7–182.9 meters) from North Carolina to the Florida Keys were gridded following the Southeast Area Monitoring and Assessment Program (SEAMAP) 1199 grid [15]. Each grid cell was one-minute latitude by one-minute longitude. The grid extended from the shoreline to approximately five nautical miles beyond the 200 m depth contour (roughly the continental shelf break). Each grid cell within the one-minute grid was coded to a bottom type of Hard Bottom (HB), Possible Hard Bottom (PH), or Not Hard Bottom (NH), based on the categorization by FWC (2001) of the SEAMAP Bottom Mapping data that intersected the grid cell. If a cell had any HB data in it, it was coded to HB regardless of any NH data in the cell. If a cell had NH and no other type of data, it was coded to NH. If a cell was not sampled, it was coded as Unknown (UN).

A variety of supplemental bathymetric layers were assimilated from the National Oceanographic and Atmospheric Administration (NOAA), SEFIS, USGS, US Navy, and NCCOS (A. David and G. Sedberry, NOAA, pers. comms.; NCCOS data available from: http://ccma.nos.noaa.gov/ecosystems/sanctuaries/south_atlantic/data/). Data were merged into a layer, clipped by the SEAMAP grid, and evaluated using surface statistics for maximum percent slope. Because the average maximum percent slope across SEAMAP cells categorized as HB by FWC (2001) was 1.45, SEAMAP cells categorized as UN were recategorized as PH if their max slope from the supplemental bathymetric sources was greater than 1.45.

Using the Coastal Relief Model (www.ngdc.noaa.gov/mgg/coastal/startcrm.htm), the habitat categorization grids were clipped by 5-fathom bins, creating depth-grids. Information was projected as UTM NAD83 Zone 17N and areas (km^2^) were assigned to clipped depth-grids using Hawth's Tools [16]. Point data were plotted at the set level for observations of speckled hind, warsaw grouper, and all sets. These data were counted within depth-grids. Analyses focused on the 30,275 depth-grids within the 25–100 fathom depth range; this encompassed the majority of observations of mature fish and was the primary area of concern with regards to barometric trauma and associated high release mortality.

Each SEAMAP depth-grid was classified as follows: ‘Known’ – A speckled hind was observed by a data source other than HBS; ‘Not suitable’ – Habitat type was ‘NH’ if no speckled hind were observed and more than 5 samples were taken in a depth-grid; ‘Probable’ – A HBS observation fell within the depth-grid or the habitat type was ‘HB’ or ‘PH’; ‘Unknown’ – Fewer than 5 negative samples and no identified habitat within the depth-grid. Observations for headboat (HBS) were treated differently due to concerns about the reliability of headboat spatial reporting. The percentage of area falling into the various habitat classifications was computed, and the proportion of these habitat classifications already contained within currently established and proposed MPAs was determined. The same process was followed for warsaw grouper.

### Geographic distribution model

Logistic regression analysis was used to develop a quantitative model predicting the spatial distribution of speckled hind and warsaw grouper. The logistic regression modeled the probability of detecting an individual within a given depth-grid as a function of gear type, depth, latitude, and habitat (Table 2). Gear type and habitat were treated as factors, and depth and latitude effects were tested in the model as continuous variables, squared terms, and factors of varying bin sizes. Because the recreational headboat logbook records contain self-reported spatial locations constrained to a 1/6°×1/6° grid, and are not necessarily reliable, we reran our models with and without this data type included to assess the effect of the headboat data on the results. The headboat logbook records make up 70% of the total observations, and thus have the greatest influence on the analysis; we also tested the exclusion of other gear types which made up more than 5% of observations (MMAP, RFOP) to ensure that our results were robust. Logistic regression analysis using a logit link was implemented using R version 2.13.2 [17]. The same modeling process was followed for both speckled hind and warsaw grouper separately. Models treated data in binary form because multiple observations were rare, and because ‘catchability’ differed among the various survey methods used. The expected grouper abundance for each habitat, depth and latitude were computed using the logistic model parameter estimates, controlling for the sampling effects of the gear.

**Table 2:** Input variables considered in logistic geographic distribution model.

Variable	Description
**lat_cont**	WGS Latitude; treated as continuous variable
**lat_sq**	Quadratic function of WGS Latitude
**lat**	WGS Latitude, treated as categorical, binned at a 1-degree resolution
**dep**	10-fathom depth bins; treated as categorical
**dep_fine**	5-fathom depth bins; treated as categorical
**dep_sq**	Quadratic function of 5-fathom depth bins
**hab**	Habitat classification: ‘HB’- hardbottom or possible hardbottom, ‘NH’- not hardbottom, ‘UN’- unknown
**gear**	Gear classification*: MARMAP, HBS, FRG, SEFIS Trap, SEFIS Video, Oculina ROV, DW-ROV, RFOP, REEF

* See text for description of data sources.

We evaluated potential models of grouper probability of occurrence including all combinations of factors. Model selection applied a cross-validation procedure in which the data were split up into training and testing sets, and a model was fit to the training set and subsequently tested on the “unseen” testing set. We employed 10-fold cross validation [18] such that the data set was randomly split into 10 groups, each group serving once as a testing set for a model trained on the other 9 groups of data. For each of the 10 folds of cross-validation, all possible models including the different combinations of factors were trained on the training set. For each model, we created a receiver operating characteristic (ROC) curve, which expresses the performance of a binary classification method such as the logistic regression used here (R ‘pROC’ library; [19]). Using the ROC curve for each model, we calculated the threshold at which the proportion of correctly classified positive observations plus the proportion of correctly classified negative observations are maximized. Using the parameters defined by each model, as well as the threshold defined by the ROC curve for each model using the training set, we then made predictions for the testing set. Model performance was calculated by creating a contingency table, which specified the rate of false positive predictions and false negative predictions for the testing set. Better performing models were those with lower false positive rates (FPR) and false negative rates (FNR). Model performance was also measured according to the reduction in Akaike's Information Criterion (AIC; [20]).

The confidence intervals around the FNR and FPR rates were quite high, due to the nature of subsetting an already sparse data set (i.e., occurrence rates of 2–7%) for the 10 fold cross-validation procedure. To further test the robustness of our model selection procedure, we repeated the cross-validation at least 10 times for each species, by re-randomizing the testing and training data sets each time and repeating the procedure. We also carried out 5-fold cross-validation, to determine whether the number of folds had any bearing on the results. Throughout these procedures, a single model for each species stood out as the model with the highest prediction power as exhibited by the lowest FNR and FPR rates. Thus, we felt justified in using the cross-validation technique to select a single best model. In the case of speckled hind, reserve protection predicted by the best predictive model and the model with the lowest AIC were presented as a range to quantify inter-model uncertainty.

### MPA Protections

To evaluate the impacts of existing and proposed spatial closures, closures were overlaid on speckled hind and warsaw grouper probability of occurrence maps. For the spatial classification model, the total area of each habitat classification contained within each MPA was summed for each stock. This was subsequently expressed as a percentage of the total area of that habitat classification within the entire SAFMC shelf-edge (25–100 fathoms) jurisdiction:
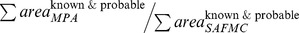
For the geographic distribution model, the probability of detection weighted by area within each depth-grid within each MPA was tallied. This was subsequently expressed as a percentage of the total area-weighted probability of detection within the entire SAFMC shelf-edge (25–100 fathoms) jurisdiction:
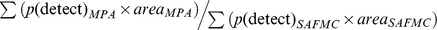
Reserve protection was also computed per unit area, allowing for comparison of tradeoffs between conservation of stock versus area closed to fishing.

## Results

### Data Sources

**Figure 2:**
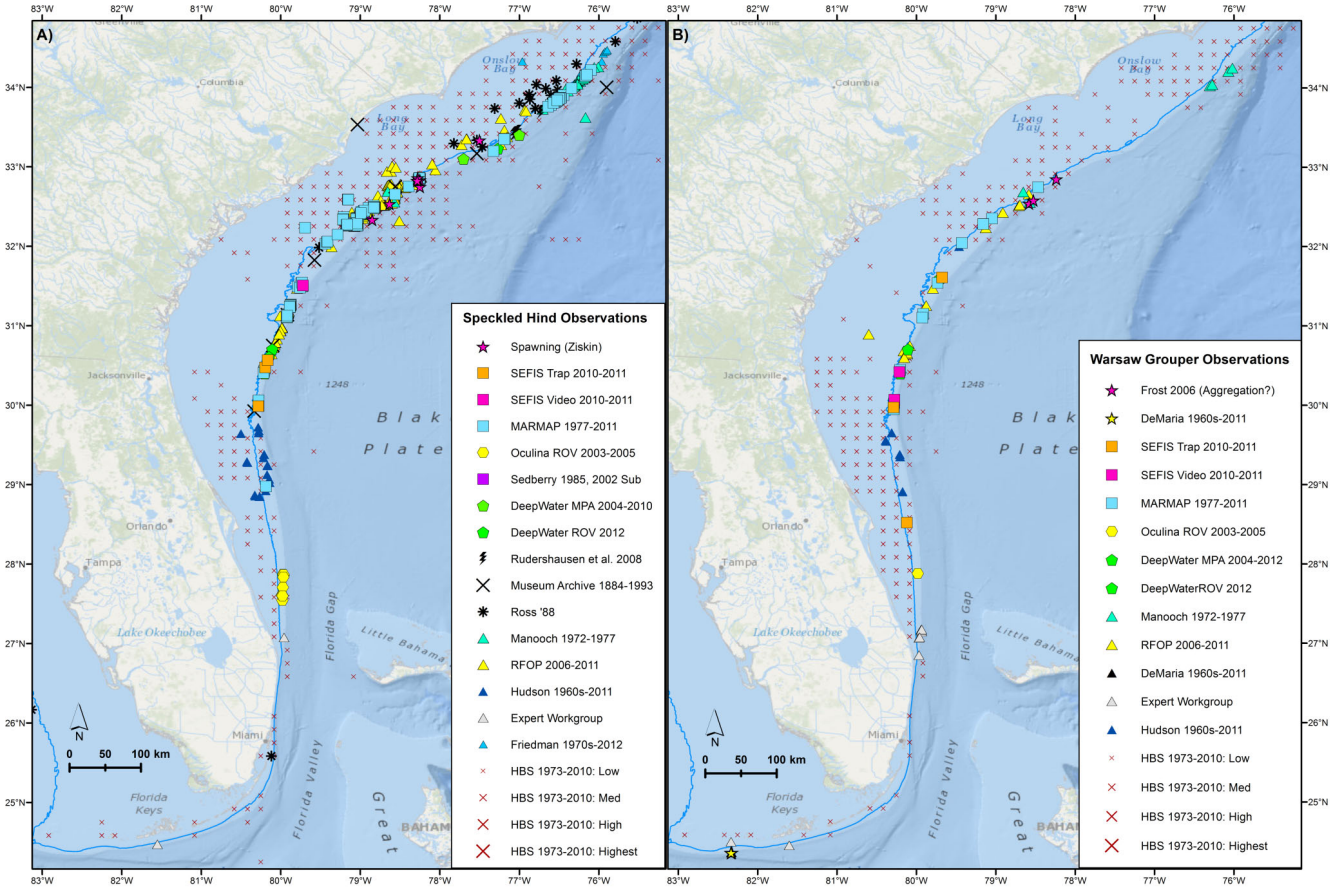
Point observations. **A**) Speckled hind and **B**) warsaw grouper encounters reported by various data sources. 25 fathom (45.7 m) bathymetric line in blue. Basemap courtesy of Esri Ocean Basemap and its partners.

**Figure 3:**
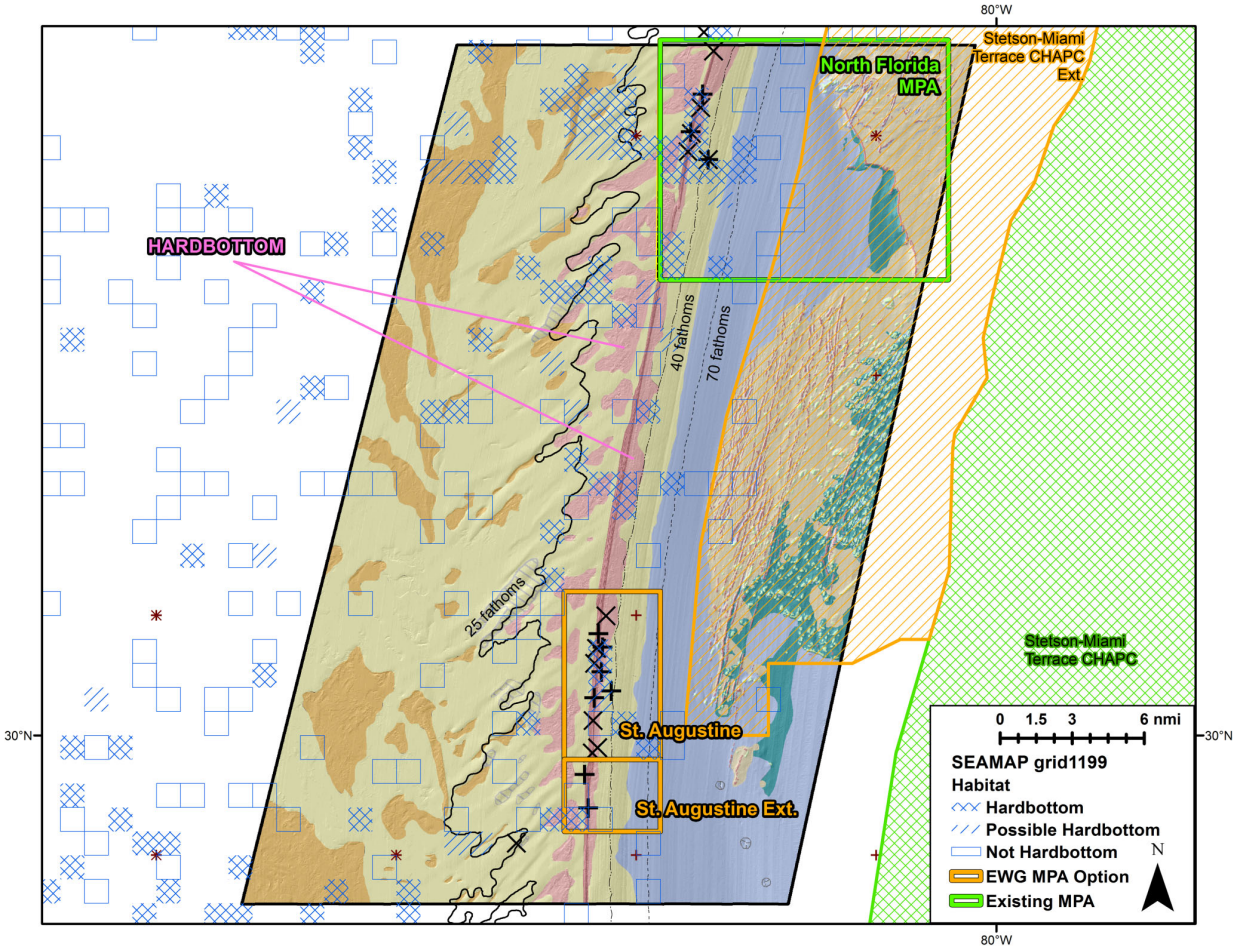
Point observations relative to habitat. Speckled hind (X) and warsaw grouper (+) encounters reported by various data sources versus habitat off Northeast Florida. Habitat data courtesy U.S. Navy, NOAA, and USGS (Andy David, NOAA, pers. comm.).

Plots of point observations of speckled hind and warsaw grouper indicated that the stocks were predominantly distributed on the shelf edge between 25–100 fathoms (45.7–182.9 meters), with concentrations in certain locations in 30–45 fathoms (54.9–82.9 m; Fig. 2). The spatial distribution of headboat observations suggested positioning inaccuracies when compared with other, more reliable, point data sources (Fig. 2). Observations were heavily concentrated in heavily-sampled areas such as hardbottom habitat features within and adjacent to the existing Northern South Carolina MPA, Edisto MPA, North Florida MPA, and Oculina Experimental Closed Area. Concentrations of observations visually corresponded to areas with hardbottom; this trend was most obvious in areas with high-resolution habitat mapping (Fig. 3).

### Spatial classification model

**Figure 4:**
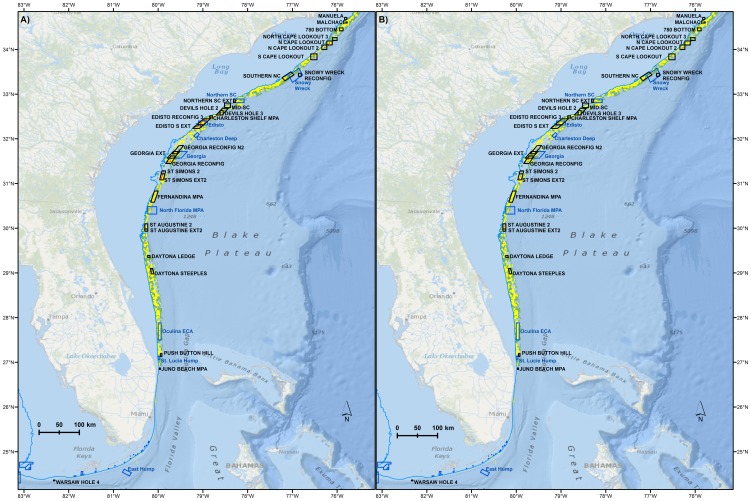
Habitat classification model output. Habitat classifications (red: ‘known’, yellow: ‘probable’, green: ‘not suitable’) for **A**) speckled hind and **B**) warsaw grouper relative to existing (blue) and proposed (black) marine protected areas. 25 fathom (45.7 m) bathymetric line in blue. Basemap courtesy of Esri Ocean Basemap and its partners.

The spatial classification modeling approach identified known and probable speckled hind habitats following a consistent hardbottom ridge that moved between depth contours of 25–100 fathoms from North Carolina to southeast Florida (Fig. 4a). Warsaw grouper were more rarely encountered, but their range also appeared to encompass more of the southern end of the SAFMC's jurisdiction (Fig. 4b). The spatial classification model indicated that of the 23,592 km^2^ of habitat between 25–100 fathoms in the SAFMC's jurisdiction, very little had been positively identified as ‘Known’ habitat (speckled hind: 329 km^2^, warsaw grouper: 76 km^2^). By contrast, the spatial classification model identifed a substantial quantity of ‘Probable’ habitat (speckled hind: 6984 km^2^, warsaw grouper: 7090 km^2^). Approximately 28% of the habitat between 25–100 fathoms was identified as unsuitable for speckled hind or warsaw grouper, and an additional 41% was unidentified.

### Geographic distribution model

Logistic regression models for probability of detection for speckled hind and warsaw grouper found latitude, habitat type, and sampling gear to be important predictors of the probability of a positive observation (Table 3). The model with the lowest AIC was not necessarily the best predictive model, per 10-fold cross-validation (Table S1). For speckled hind, the model with the lowest AIC included the gear effect, latitude as a categorical variable, depth as categorical variable, and habitat. The false positive rate (FPR ± 1 S.D.) for this model was 0.248±0.166 and the false negative rate (FNR) was 0.269±0.181. The speckled hind model with the highest predictive power as assessed by cross-validation (i.e., the lowest summed FPR and FNR) included only gear, latitude as a continuous variable, and depth as a squared term. For this model, the FPR was 0.246±0.162 and the FNR was 0.234±0.148, and the deviance explained was 36.8 percent. When excluding the headboat logbook data for the speckled hind model, results in terms of model selection via both AIC and cross-validation were exactly the same, but FPR and FNR ratios for the best predictive model were increased to 0.255 and 0.357, respectively. This indicates that the information from the headboat sector in regards to distribution of specked hind was in agreement with information from other gear types, and that inclusion of headboat data improved model performance. Exclusion of other gear types also yielded similar results in terms of model selection, and therefore headboat data and all other gear types were retained for the final results. For warsaw grouper, probability of occurrence was only modeled for latitudes greater than 28 degrees north, because observations south of this point were extremely scarce (only 4 positive observations out of 11,146 data points were available). For warsaw grouper, the same model produced both the lowest AIC value and the highest predictive power (FPR = 0.282±0.227, FNR = 0.330±0.281). This model included gear effect, latitude as a categorical variable, and a squared depth term, and explained 10.8 percent of the variability in the probability of detection.

**Table 3:** Logistic regression model maximum likelihood parameter estimates for speckled hind and warsaw grouper probability of detection, with deviance explained (i.e. percent variability explained by inclusion of additional variable).

Speckled hind (model 5)					
Parameter	Estimate	Std. Error	z value	Pr(>|z|)	Deviance explained
Intercept	−20.00	0.56	−35.94	<0.001	-
gear FRG	0.53	0.37	1.44	0.15	2.8%
gear HBS	0.67	0.27	2.52	0.01	-
gear MMAP	−1.81	0.28	−6.58	<0.001	-
gear Oculina	2.36	0.44	5.36	<0.001	-
gear Reef	0.61	0.65	0.95	0.34	-
gear RFOP	−0.62	0.28	−2.23	0.03	-
gear SEFIS-trap	−2.55	0.64	−4.00	<0.001	-
gear SEFIS-video	−2.64	0.76	−3.48	<0.001	-
lat_cont	0.59	0.02	38.81	<0.001	33.1%
dep_sq	0.00	0.00	−7.21	<0.001	0.9%
					**36.8%**

Warsaw grouper (model 14)					
Parameter	Estimate	Std. Error	z value	Pr(>|z|)	Deviance explained
Intercept	−2.92	0.66	−4.44	<0.001	-
gear FRG	1.01	0.85	1.18	0.24	8.4%
gear HBS	0.29	0.62	0.46	0.65	-
gear MMAP	−2.33	0.68	−3.43	<0.001	-
gear Oculina	−12.70	334.00	−0.04	0.97	-
gear RFOP	−1.17	0.66	−1.79	0.07	-
gear SEFIS-trap	−1.87	0.84	−2.24	0.03	-
gear SEFIS-video	−1.35	0.79	−1.71	0.09	-
lat [29], [30]	−0.74	0.79	−0.93	0.35	2.5%
lat [30], [31]	0.39	0.43	0.90	0.37	-
lat [31], [32]	−0.16	0.39	−0.40	0.69	-
lat [32], [33]	−0.93	0.25	−3.70	<0.001	-
lat [33], [34]	−1.77	0.47	−3.79	<0.001	-
lat [34], [35]	−0.52	0.41	−1.26	0.21	-
dep_sq	0.00	0.00	0.47	0.64	0.02%
					**10.92%**

**Figure 5:**
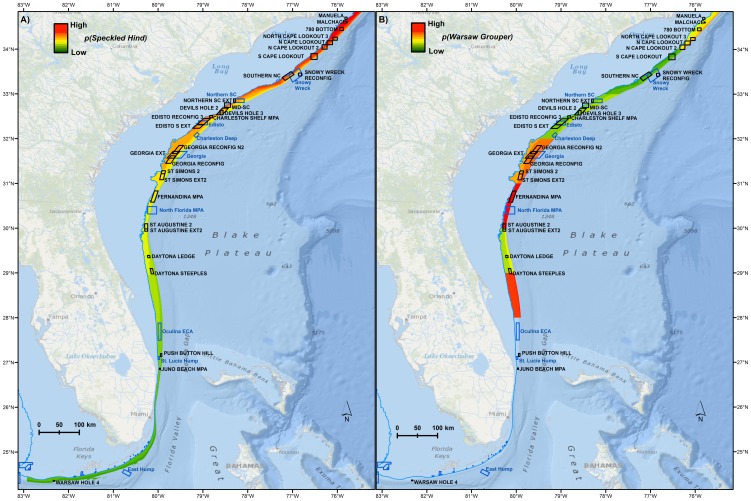
Geographic distribution model predictions. Probability of encounter with **A**) Speckled hind and **B**) warsaw grouper relative to existing (blue) and proposed (black) marine protected areas. 25 fathom (45.72 m) bathymetric line in blue. Basemap courtesy of Esri Ocean Basemap and its partners.

Maps of probability of occurrence across space for the two species were produced based on the best model as defined by the highest prediction power. Generally, speckled hind probability of occurrence increased with latitude, whereas occurrence of warsaw grouper decreased with latitude (Fig. 5). Speckled hind distributions were more shallow (e.g., 25–50 fathoms) relative to warsaw grouper, which were more evenly distributed across the 25–100 fathom range. Warsaw grouper probability of occurrence was highest off the coast of Georgia, and also between 28 and 29 degrees N, off the coast of Florida. Anecdotal information [21] suggests relatively high encounter rates with warsaw grouper south of Cape Canaveral, but we did not have enough data coverage to assess occurrence in this region.

### MPA Protections

**Figure 6:**
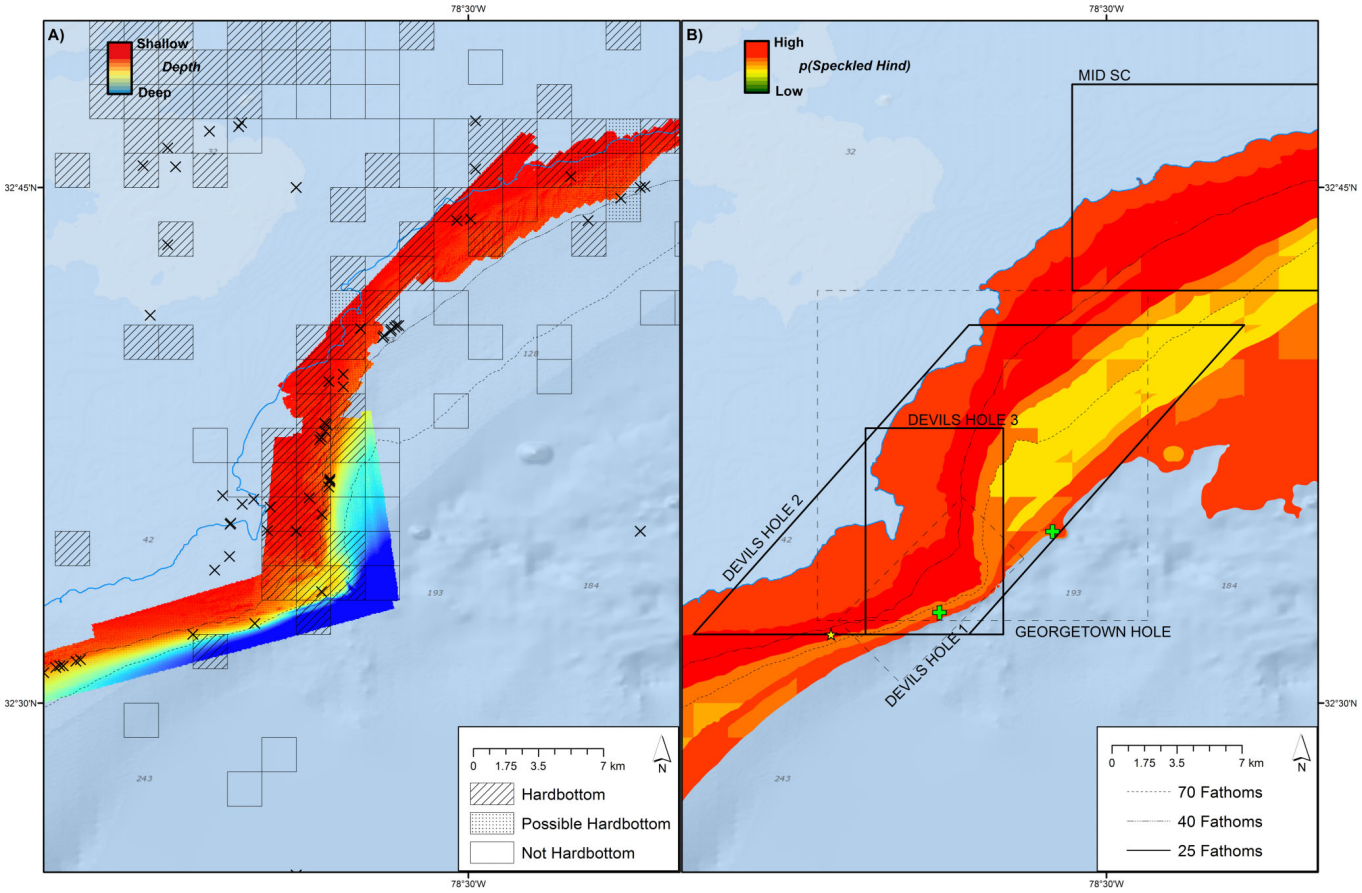
Point and spawning observations. **A**) Point observations of speckled hind (X) and warsaw grouper (+) relative to bathymetry and **B**) anecdotal spawning or aggregation observations of speckled hind (yellow star) and warsaw grouper (green crosses) relative to speckled hind geographic distribution model output and rejected (dashed lines) and proposed (solid lines) marine protected areas east of Murrell's Inlet, SC. Basemap courtesy of Esri Ocean Basemap and its partners.

Proposed and existing MPAs varied in the estimated level of protection they provided to speckled hind and warsaw grouper habitats and the percent of grouper estimated to be contained within their boundaries (Table 4). Dynamic hardbottom habitats appeared to yield the highest conservation benefit per unit area and some also contained observed spawning condition fish (Fig. 6). The highest percentage of known habitat for speckled hind was reflective of the concentration of scientific sampling around the proposed Edisto Reconfig 3, Edisto S Ext, and existing Edisto MPA. Likewise, for warsaw grouper, the highest percentage of known habitat was contained in the proposed Georgia Reconfig, Edisto S Extension, Fernandina MPA, and the existing North Florida MPA. The highest estimated percentage of known and probable habitat for speckled hind and warsaw grouper was contained within the proposed Oculina Coral Habitat of Particular Concern (CHAPC) Extension, the existing Oculina Experimental Closed Area (ECA), and the proposed Edisto Reconfig 3 and Fernandina MPA sites.

**Table 4:** Evaluation of existing (lower case) and proposed (italicized, all caps) no-take marine reserves for speckled hind and warsaw grouper relative to coverage of viable habitats and percent of grouper protected, as predicted by geographic distribution models.

		SPECKLED HIND HABITAT SUITABILITY				WARSAW GROUPER HABITAT SUITABILITY		
Name	Area (km^2^)	Known	Known & Probable	% Stock [X-val]	% Stock [AIC]	Known	Known & Probable	% Stock
**NORTH CAROLINA**								
*780 BOTTOM*	56.9	0.00%	0.74%	0.99%	0.36%	0.00%	0.75%	0.46%
*MANUELA WRECK*	25.5	0.00%	0.00%	0.01%	0.%	0.00%	0.00%	0.00%
*MALCHACE WRECK*	6.4	0.00%	0.14%	0.14%	0.05%	0.00%	0.14%	0.05%
*N CAPE LOOKOUT 2*	114.8	2.63%	0.83%	1.1%	0.38%	2.44%	0.83%	0.62%
*N CAPE LOOKOUT NC*	110.9	2.33%	1.09%	1.13%	0.39%	0.00%	1.09%	0.62%
*NORTH CAPE LOOKOUT 3*	68.4	0.72%	0.20%	0.57%	0.17%	1.76%	0.20%	0.34%
*S CAPE LOOKOUT NC*	187.5	1.80%	1.07%	1.4%	1.4%	0.00%	1.02%	0.26%
*SOUTHERN NC*	229.9	0.74%	1.69%	1.53%	1.68%	0.00%	1.73%	0.34%
**SOUTH CAROLINA**								
Charleston Deep	66	0.00%	0.09%	0.28%	0.63%	0.00%	0.09%	0.31%
*CHARLESTON SHELF MPA*	34.8	3.58%	0.55%	0.29%	0.68%	0.00%	0.44%	0.16%
*DEVILS HOLE 2*	208.3	6.47%	1.72%	1.12%	2.11%	1.81%	1.72%	0.78%
Edisto	191.4	9.16%	1.85%	1.15%	2.88%	1.24%	1.65%	0.79%
*EDISTO RECONFIG 3*	208.7	20.09%	2.96%	1.52%	4.03%	2.39%	2.45%	0.93%
*EDISTO S EXT*	130.6	10.08%	1.10%	0.8%	2.07%	10.14%	0.88%	0.51%
*DEVILS HOLE 3*	69.4	2.60%	0.99%	0.41%	0.88%	0.70%	0.99%	0.26%
*MID SC MPA*	138.7	2.69%	0.69%	0.52%	1.1%	0.15%	0.63%	0.30%
Northern SC	173.2	3.81%	1.46%	1.02%	1.99%	2.52%	1.36%	0.76%
*NORTHERN SC EXT*	32.5	3.35%	0.22%	0.17%	0.28%	0.00%	0.10%	0.08%
**GEORGIA**								
Georgia	262.9	0.00%	0.76%	0.39%	1.17%	0.00%	0.78%	1.34%
*GEORGIA MPA RECONFIG*	204.7	4.89%	2.38%	0.97%	3.41%	11.20%	2.32%	1.87%
*GEORGIA EXT*	236.6	0.00%	2.01%	1.38%	4.01%	8.20%	2.14%	2.52%
*GEORGIA RECONFIG N2*	192.5	0.00%	1.57%	1.11%	3.31%	0.00%	1.60%	1.95%
*ST SIMONS 2*	58.6	3.27%	0.19%	0.32%	0.89%	2.23%	0.06%	0.66%
*ST SIMONS EXT2*	117.4	5.26%	1.09%	0.42%	1.45%	5.21%	0.98%	0.96%
**NORTHEAST FLORIDA**								
*FERNANDINA MPA*	221.1	1.11%	2.46%	0.48%	1.13%	7.02%	2.57%	2.80%
North Florida	354.9	2.46%	1.86%	0.36%	0.96%	4.53%	1.88%	2.56%
Oculina Bank CHAPC (excluding ECA)	753.7	0.00%	1.03%	0.11%	0.08%	0.00%	1.05%	2.05%
*OCULINA BANK CHAPC EXTENSION (excluding DAYTONA STEEPLES and DAYTONA LEDGE)*	627.7	0.46%	2.29%	0.34%	0.32%	0.00%	2.33%	2.33%
Oculina ECA	279.2	3.79%	3.19%	0.11%	0.28%	2.61%	3.26%	*Not eval.*
*DAYTONA STEEPLES*	68.9	1.51%	0.76%	0.1%	0.12%	0.00%	0.70%	0.55%
*DAYTONA LEDGE*	28.4	1.79%	0.31%	0.05%	0.08%	7.76%	0.31%	0.22%
*ST AUGUSTINE 2*	83.1	1.92%	0.66%	0.15%	0.45%	9.17%	0.62%	1.05%
*ST AUGUSTINE EXT2*	35.6	0.87%	0.21%	0.07%	0.05%	1.04%	0.18%	0.20%
**SOUTHEAST FLORIDA**								
*FKNMS SPAs & Ers*	246.7	*Not evaluated*				*Not evaluated*		
*JUNO BEACH MPA*	9.2	0.00%	0.00%	0.%	0.%	0.54%	0.01%	*Not eval.*
*PUSH BUTTON HILL*	24.4	0.00%	0.27%	0.01%	0.01%	3.61%	0.30%	*Not eval.*
St. Lucie Hump	24.4	0.00%	0.19%	0.01%	0.04%	1.01%	0.20%	*Not eval.*
*WARSAW HOLE 4*	6.2	0.00%	0.00%	0.%	0.%	3.08%	0.03%	*Not eval.*

Output range for cross-validation best predictor (X-val) and best-fitting (AIC) models for speckled hind provided to characterize uncertainty. Note that geographic distribution model was unable to resolve probabilities south of 28′ latitude for warsaw grouper.

**Note:** Assumes CHAPC no-anchoring provision results in 50% efficiency at eliminating bycatch mortality. Warsaw grouper percent stock estimates not generated south of 28° latitude. Oculina Bank CHAPC evaluation excludes Experimental Closed Area (ECA). Oculina Bank CHAPC Extension evaluation excludes Daytona Steeples and Daytona Ledge.

The best predictive model for speckled hind (per cross-validation) suggested the highest estimated percentage of the stock was contained in the proposed Edisto Reconfig 3 and three proposed extensions/reconfigurations of the Georgia MPA (Table 4). The best-fitting model for speckled hind (per AIC) indicated the highest estimated percentage of the stock was contained within the proposed Southern NC, Edisto Reconfig 3, and South Cape Lookout MPAs. The highest estimated percentage of warsaw grouper stock was contained within the proposed Georgia Extension, the existing North Florida MPA, and the proposed Fernandina closed area.

**Figure 7:**
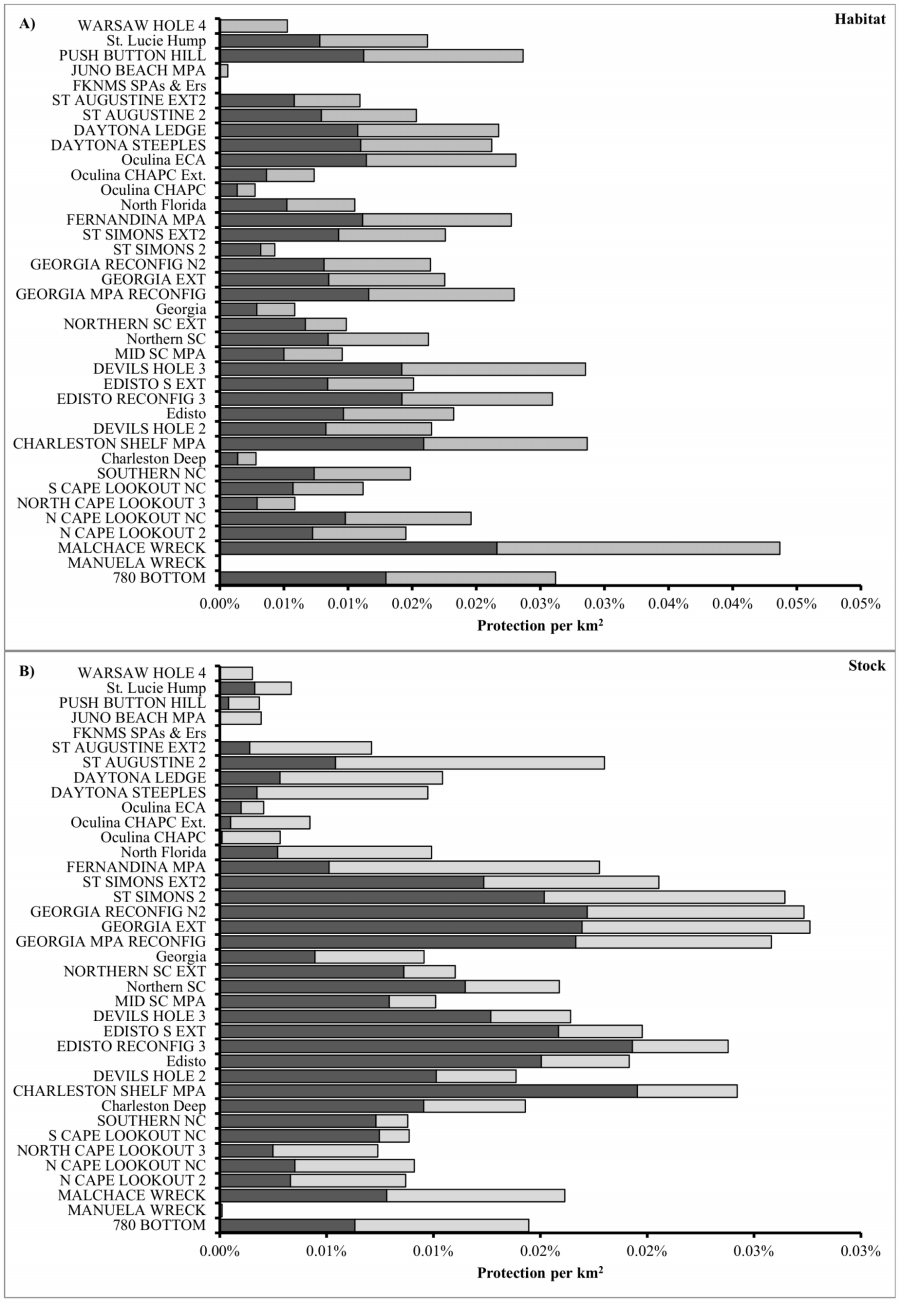
No-take marine reserve protection per unit area. Percent of **A**) known and probable habitat and **B**) speckled hind (black) and warsaw grouper (light gray) stock per square kilometer.

The most efficient reserves for the two stocks together, based on spatial classification model predictions, were the proposed Malchace Wreck and Devil's Hole 3 MPAs (Fig. 6), reconfigurations and extensions of the Edisto MPA, Push Button Hill, and the existing Oculina ECA (Fig. 7A). The most efficient reserves for both stocks together, based on geographic distribution model predictions, were the Georgia Extension, the Georgia Reconfig N2, the St. Simons 2, the Georgia Reconfig, Charleston Shelf MPA, and Edisto Reconfig 3 (Fig. 7B). The most efficient reserves for speckled hind, based on the spatial classification model, were the Malchace Wreck, Charleston Shelf, Edisto Reconfig 3, and 780 Bottom (Fig. 7A, black fill). The most efficient reserves for speckled hind, based on the geographic distribution model, were the Charleston Shelf, Edisto Reconfig 3, and Georgia Reconfig N2 (Fig. 7B, black fill). The most efficient reserves for warsaw grouper, based on geographic distribution model predictions, were the Malchace Wreck, Devil's Hole 3, Charleston Shelf, and 780 Bottom (Fig. 7A, gray fill). The most efficient reserves for warsaw grouper, based on geographic distribution model predictions, were the St. Augustine 2, Fernandina, and St. Simons Ext2 (Fig. 7B, gray fill).

Overall, our models estimated the 2,352 km^2^ of existing deep-water MPAs and CHAPCs covered 19% of the ‘Known’ habitat and 10% of the ‘Known & Probable’ habitat for speckled hind, and protected between 3–8% of speckled hind between 25–100 fathoms. For warsaw grouper, 12% of ‘Known’ habitat and 10% of ‘Known & Probable’ habitats were estimated to be within existing MPAs, and 8% of warsaw grouper between 25–100 fathoms (north of 28° latitude) were estimated to be protected. The 3,093 km^2^ of non-overlapping (e.g., excluding Devil's Hole 2 and Georgia Ext.) proposed MPA and CHAPC options were estimated to cover 72% of ‘Known’ and 24% of ‘Known & Probable’ speckled hind habitats, containing between 15–25% of speckled hind between 25–100 fathoms. The proposed closed area options were estimated to cover 68% of ‘Known’ and 23% of ‘Known & Probable’ warsaw grouper habitats, containing approximately 18% of warsaw grouper between 25–100 fathoms (north of 28° latitude).

## Discussion

Conclusions regarding the status, distribution, and impacts of spatial protection for speckled hind and warsaw grouper remain uncertain. This uncertainty stems from a lack of available habitat data on scales relevant to the habitat usage of these species, as well as the rarity of the stocks. Records of warsaw grouper and speckled hind in commonly used fishery-dependent and fishery-independent data sources were limited. When data were available, catch location was often unavailable or very coarse in resolution, and thus linking these point observations to specific habitat features would be challenging even if improved habitat data were available. Prior to the early 1990s, speckled hind and warsaw grouper were not identified to species in the commercial logbooks, and a harvest prohibition began in 1994. As such, conclusions that might be drawn about the distribution of the stock from post-1994 data suffer from biases for under-representation due to the disincentive to retain the fish, and incentives to misidentify the fish if kept and sold. Depth was unavailable for most datasets. For data sources with depth, samples were most frequent from depths beyond 160 ft (48.7 m), but sampling/fishing pressure were much higher at shallower depths.

To control for all these confounding factors, and attempt to overcome the challenges associated with the lack of fine-scale habitat data, we consolidated a broad variety of fishery-independent, fishery-dependent, and anecdotal data sources. All data sources appeared to tell a consistent story regarding the habitats, depths, and latitudinal distribution of speckled hind and warsaw grouper. Both stocks were heavily associated with the shelf-edge between 25–100 fathoms (45.7–182.9 meters) on hardbottom habitats. Neither species was found with any frequency in the mostly mud-bottom habitats on the shelf-edge north of Cape Hatteras, North Carolina. Speckled hind were most commonly observed south of Cape Hatteras, North Carolina to northeast Florida. Warsaw grouper were most commonly observed from South Carolina to northeast Florida.

### Modeling approaches

Using a suite of qualitative and quantitative approaches, we were able to generate reasonable estimates for the occurrence of speckled hind and warsaw grouper across space, and were therefore able to estimate the conservation benefits of existing and proposed marine protected areas. The spatial classification model provided a comprehensive semi-quantitative method for assimilating all available observation and habitat data. The spatial classification model appeared to provide useful predictions; following model development, additional point data were obtained from [22] and the NOAA Deepwater ROV 2012 survey. These new point observations were located within model-identified ‘Known’ and ‘Probable’ habitats. The spatial classification model provides information for warsaw grouper south of 28°N latitude, where data was too scarce for useful geographic distribution model predictions. Anecdotal information suggests the southeast Florida shelf-edge may have been historically important warsaw grouper habitat in the western North Atlantic Ocean, and this impression is supported by the spatial classification model. The spatial classification model, due to its simplistic nature, was able to provide broader predictive coverage than the more rigorous distribution model. A weakness of this simplified modeling approach was the coarse designation of habitat within each SEAMAP sampling grid (see Fig. 3). Any hardbottom or possible hardbottom within one of these one arc-minute squares would result in the whole grid (∼3 km^2^) being categorized as suitable habitat. This approach fails to consider factors that might make certain hardbottom habitats more ‘suitable’ than others – for example, speckled hind and warsaw grouper may prefer high-relief rocky reef near reef slopes, but the available data was too coarse to distinguish this preference. Additionally, the coarse categorization of the habitat data might overestimate the total ‘Known’ and ‘Probable’ habitat; however, this bias was systematic, and suitable habitat within reserves was expressed as a proportion of the total suitable habitat within the entire SAFMC jurisdiction. By expressing suitable habitat as a proportion, inter-reserve comparisons should be valid unless habitats in certain areas were less effectively categorized than others, or if certain areas featured higher concentrations of a type of hardbottom more preferred by the stock.

A geographic distribution model based on several predictor variables – gear type, latitude, and depth – was able to explain approximately 10% of the variability in warsaw grouper probability of detection and over one-third of the variability in speckled hind probability of detection. A comparable modeling attempt with a data-rich species, using a comprehensive fishery-independent survey, yielded similar results in terms of total variability explained by depth and geographical bins [23]. In this study, the presence of red snapper was modeled as a function of depth and longitude bin, among other sampling factors such as gear type. Despite the relatively high occurrence rate of red snapper (fish present at 507 of 3102 sites), and the reliability of the synoptic fishery-independent survey, depth and longitude explained only 8.5% and 6.6% of the variability in presence of red snapper after the removal of sampling artifacts. In our speckled hind model, latitude and depth explained over twice as much deviance as the red snapper model, after the removal of gear effects. This comparison suggests the overall low explanatory power of our models is due to the lack of appropriate explanatory variables, rather than a lack of reliability in the data sets used. For warsaw grouper, the limited model deviance explained by latitude and depth was likely due to the very low occurrence rates observed for this species.

While only a modest amount of the variability in detections was explained, the geographic distribution models still performed fairly well in terms of their predictive ability. Rates of false positive identifications and false negative identifications were below 25% for the best speckled hind model, and for warsaw grouper, false identification rates were only slightly higher (28–33% for the best model). Given that false positive and false negative identification rates would be about 50% for a completely random model, based purely on chance, our quantitative models reduced the rate of incorrectly predicted observations by about one-half.

The detailed cross-validation procedure carried out in this study yielded some interesting results in terms of model performance and selection. For both species, gear effects and latitude were the primary drivers for probability of detection. Once these two factors had been accounted for, additional factors had minimal impact on the predictive efficiency. Confidence intervals around the false identification rates suggested that all models including at least gear and latitude as factors were statistically equivalent in terms of prediction power. The suite of models including both gear and latitude effects did, however, differ widely in their AIC values, and in the predictions that resulted from these models. In other words, while many of the candidate models had similar predictive capabilities as tested by cross-validation, the ultimate outputs in terms of the percentage of grouper protected by each MPA were quite sensitive to the model chosen (see Table 4). Because the procedure for selecting the “best” model (e.g., AIC versus cross-validation) had an important influence on the final results for speckled hind, both models were presented, to more effectively capture the uncertainty associated with model predictions.

While the modeling approaches used here give us some confidence in identifying potential areas for improved protection of the study species, ultimately our ability to definitively distinguish the benefits of these area closures and to map these species with increased confidence will require additional data. It was surprising to find that including a habitat factor in the geographic distribution model did not improve model performance, particularly given that both speckled hind and warsaw grouper are found almost exclusively on hard bottom habitats. We believe the best explanation for the apparent inutility of the habitat data is that important habitat features are usually present at scales well below the resolution of the habitat data. For example, speckled hind and warsaw groupers are well-known to inhabit wrecks, but these small features will often be located within large areas of no hard bottom habitat. Thus, additional high-resolution habitat mapping may be necessary to enhance the predictive utility of this variable. As previously discussed, simple ‘hardbottom’ habitat may be an inadequate classification scheme for these stocks, which may require particular hardbottom features such as ledges, pinnacles, or other fine-scale features beyond the resolution of currently available data. As such, estimates of reserve protection should be validated with empirical observations demonstrating that suitable habitat is present within the proposed closure area. Our findings emphasize the need for detailed geomorphological maps using multibeam or sidescan technologies backed with groundtruthing along the entire southeastern U.S. shelf edge. Around 41% of the shelf-edge remains uncategorized with regards to habitat type, and the resolution of the habitat categorizations in the areas that have been studied is insufficient for many management needs. Additionally, other variables, such as physical oceanographic metrics, or location in reference to features such as channels may also be useful in predicting occurrence of grouper species [24].

### MPA Recommendations

MPAs have been endorsed as fisheries management tools that, when used in conjunction with traditional management, may help ensure sustainability of intensely exploited regional fisheries resources [25]. Theory suggests that buildup of fish biomass, density, and average size in no-take MPAs due to reduced exploitation (e.g., [26]–[28]) will result in density-dependent emigration of adult fish across MPA boundaries [29]. Additionally, larval production should be amplified by the larger, older population within the MPA due to its increased spawning stock biomass [30], [31]. The advection of these eggs and larvae by ocean currents may enhance recruitment in fishable areas [29].

Over the past two decades, there has been much scientific discussion regarding the percent of the stock that should be protected by a MPA to provide benefits such as reduced risk of overexploitation, restoration of natural community dynamics, increased spawning stock biomass, and maximization of yield through spillover of adult biomass and larval recruits. A meta-analysis of percent closure recommendations indicated a consensus that between 20–40% of the stock should be protected unless it is heavily exploited outside the MPA system ([32]; Fig. S1). The exact amount of area or stock that should be protected will depend on the specific objectives of the MPA, and will balance the biology and status of the stocks in need of protection with the regulations that exist outside of the MPA [33]. As such, there is no ‘one size fits all’ answer for the appropriate size, scale, or number of MPAs needed [33].

For the specific case of speckled hind and warsaw grouper protections, a primary goal for spatial protection would be to supplement the existing prohibition of harvest with spatial closures to reduce bycatch mortality [10], [21]. As such, MPAs would be most effective if located at sites where bycatch mortality is highest. Those sites would be in deep water, at the intersection of relatively high stock concentrations and high fishing pressure for associated species. Our analysis assumes these MPAs would eliminate bycatch mortality for all but the CHAPCs, which are assumed to reduce bycatch mortality by 50%. Poaching or fishing activities which violated the assumption of no bycatch mortality of speckled hind or warsaw grouper would invalidate the conclusions presented in this manuscript. MPAs would be most effective if scaled to the natural movements of the fish [34], [35], with a sufficient buffer to prevent the redistribution of fishing pressure on the edges of the reserve from offsetting the benefits of protection at the core. As these species do not exist in isolation, it is important that reserves designed for stock recovery also consider ecosystem processes that may be critical to their life history, including critical habitats and the scales of movement of their prey species. Designation of large shelf-edge MPAs would protect spawning aggregations of many species, allow ecosystem recovery, and minimize perimeter-to-area ratio so that loss of fish to the outside that might dilute the benefits of the MPA [25], [35].

Fish stock spatial dynamics—including preferential habitat utilization, movements and migratory behaviors—play a critical role in determining how fishing pressure will impact the stock, and result in fish stocks being heterogeneously distributed throughout the oceans [36]–[41]. Our meta-analysis of available fishery-dependent, fishery-independent, and anecdotal data told a consistent story with regards to the hardbottom obligate habitat preferences of speckled hind and warsaw grouper. Coupling that information with available habitat mapping and depth-grid specific computations of probability of encounter, we have provided some guidance regarding areas of higher concentration for these stocks. Use of point observations alone to guide reserve selection could lead to overly optimistic conclusions regarding the level of protection the stock is receiving. We have attempted to control for this bias using the spatial classification and grouper distribution models described above. The spatial classification model provides broad geographic coverage and incorporates information from all spatial data sources. The geographic distribution model controls for sampling biases and provides predictive utility for the percent stock occurring within various spatial closure alternatives. Future analyses should attempt to evaluate hydrographic linkages between MPA sites in the context of larval connectivity [24], [42], and to identify biogeomorphic features that may serve as spawning aggregation locations [43]–[45]. The fishery benefits of an MPA will be most fully realized if the MPA contains spawning habitats, especially where these habitats serve as a source for other suitable habitats. If spawning aggregation sites are outside of the reserves and are subject to bycatch mortality, many of the potential benefits of spatial protection will be undermined and objectives of population recovery will not be achieved.

Selection of MPAs containing known and probable habitats for both speckled hind and warsaw grouper would be a reasonable approach towards enhancing the protection of these stocks from bycatch mortality. Our analysis suggests that the most efficient closures would be those of reasonable size (>10–20 km^2^) that are sited in areas with high concentrations of quality habitat and high probabilities of encounter for each stock. Within the effective domain of the logistic model (speckled hind: 34°N to 26°S, warsaw grouper: 34°N to 28°S; 45.7–182.9 meters depth for both stocks), the probability of detection with gear effects removed are theoretically proportional to abundances. Thus, the sum of depth-grid cell probabilities within a given MPA divided by the sum of all SAFMC depth-grid probabilities may provide a reasonable estimate of the proportion of the grouper contained within the MPA, keeping in mind the uncertainties described above.

Less overall area would need to be closed to achieve the same level of estimated protection if the spatial protections are preferentially selected based on their predicted protection per unit area. There will likely be tradeoffs between distributing the socioeconomic impacts of spatial protection among fishermen from various coastal states; however, the greatest reductions in bycatch mortality will be realized by closing where fishing pressure for associated stocks is highest, unless this causes redistribution of fishing pressure onto adjacent areas where concentrations of warsaw grouper and speckled hind are even higher. In general, larger MPAs or MPAs closer to population centers are predicted to have the greatest economic impacts and lowest compliance rates; however, these MPAs could also provide the greatest proportional reduction in bycatch mortality. Given that all exploited stocks in the SAFMC are managed by annual catch limits, effort shifting may allow fishermen to compensate for spatial closures, and potential reductions in harvest may be offset unless core harvest locations are within the implemented MPA.

### Overall recommendation for management

Implementation of spatial closures for speckled hind and warsaw grouper should apply adaptive management principles when possible [46]. Adaptive management modifies management practices and policies to be more successful when new science, socioeconomic information or lessons learned from previous management actions indicate that practices could be made more efficient. For spatial closures such as those discussed in this study, monitoring and evaluating, testing assumptions, and generating learning opportunities are important aspects of adaptive management. Any MPAs implemented will not exist in a vacuum, and research should be conducted to understand the level of protection afforded to the stocks by the reserves and to better describe stock status. As further information emerges regarding ecosystem conditions, fishing operations, community structures, or other social, ecological, or governance factors, MPAs could be modified, added, or removed to best address management needs. Dynamic MPA management would benefit most from improved resolution on hardbottom identification and increased fishery-independent sampling over a broader geographic range using appropriate gears. A special emphasis on building a long-term robust time series of population abundance data for both stocks to allow for an updated stock assessment is also recommended.

## Supporting Information

Figure S1 **Meta-analysis of recommendations for percent closure recommendations from various peer-reviewed sources for yield maximization and reduction in risk of overfishing (see NRC 2001 for citations).**
(TIF)

Table S1 **List of candidate models to predict probability of occurrence of specked hind and warsaw grouper, with their associated AIC values and false positive (FPR) and false negative (FNR) identification rates.** Bold values denote the best model in terms of either AIC or combined FPR and FNR. Standard deviations quantify the variance around the FPR and FNR resulting from the 10 different test data sets in the cross-validation procedure.
(DOCX)
