# Supplementary material for: Spatial Distribution and Conservation of Speckled Hind and Warsaw Grouper in the Atlantic Ocean off the Southeastern U.S
Source: PLoS One. 2013 Nov 19;8(11):e78682. doi: 10.1371/journal.pone.0078682 (PMC3834121; doi:10.1371/journal.pone.0078682)
Supplement: Table S1 — List of candidate models to predict probability of occurrence of specked hind and warsaw grouper, with their associated AIC values and false positive (FPR) and false negative (FNR) identification rates. Bold values denote the best model in terms of either AIC or combined FPR and FNR. Standard deviations quantify the variance around the FPR and FNR resulting from the 10 different test data sets in the cross-validation procedure. (DOCX) [file pone.0078682.s002.docx]

**Table S1.** List of candidate models to predict probability of occurrence of specked hind and warsaw grouper, with their associated AIC values and false positive (FPR) and false negative (FNR) identification rates. Bold values denote the best model in terms of either AIC or combined FPR and FNR. Standard deviations quantify the variance around the FPR and FNR resulting from the 10 different test data sets in the cross-validation procedure.

|  | Speckled hind | | | | |  | Warsaw grouper | | | | |
| --- | --- | --- | --- | --- | --- | --- | --- | --- | --- | --- | --- |
| Model | AIC | Δ AIC | FPR (+/- SD) | FNR (+/- SD) | FPR+FNR |  | AIC | Δ AIC | FPR (+/- SD) | FNR (+/- SD) | FPR+FNR |
| gear | 10117.0 | 3827.4 | 0.457 (0.284) | 0.379 (0.319) | 0.837 |  | 1277.1 | 17.3 | 0.284 (0.240) | 0.428 (0.350) | 0.712 |
| gear, dep_sq | 7792.4 | 1502.8 | 0.351 (0.226) | 0.257 (0.137) | 0.608 |  | 1285.8 | 26.0 | 0.299 (0.238) | 0.453 (0.376) | 0.751 |
| gear, lat_sq | 6836.8 | 547.3 | 0.247 (0.157) | 0.238 (0.149) | 0.486 |  | 1262.1 | 2.3 | 0.321 (0.237) | 0.314 (0.268) | 0.635 |
| gear, lat_cont | 6780.5 | 490.9 | 0.252 (0.161) | 0.232 (0.146) | 0.484 |  | 1262.1 | 2.3 | 0.325 (0.239) | 0.314 (0.268) | 0.639 |
| gear, lat_cont, dep_sq | 6723.4 | 433.8 | 0.246 (0.162) | 0.234 (0.148) | **0.481** |  | 1264.0 | 4.2 | 0.325 (0.24) | 0.314 (0.268) | 0.638 |
| gear, lat_cont, hab | 6690.9 | 401.3 | 0.253 (0.161) | 0.236 (0.156) | 0.489 |  | 1265.6 | 5.8 | 0.315 (0.239) | 0.354 (0.279) | 0.669 |
| gear, lat_cont, hab, dep | 6492.1 | 202.5 | 0.237 (0.155) | 0.320 (0.210) | 0.557 |  | 1271.3 | 11.5 | 0.316 (0.237) | 0.372 (0.264) | 0.688 |
| gear, lat_cont, lat_sq | 6516.8 | 227.2 | 0.265 (0.193) | 0.284 (0.191) | 0.549 |  | 1264.1 | 4.3 | 0.318 (0.232) | 0.335 (0.263) | 0.653 |
| gear, lat_cont, lat_sq, hab | 6452.5 | 162.9 | 0.282 (0.197) | 0.258 (0.178) | 0.540 |  | 1267.2 | 7.4 | 0.313 (0.224) | 0.378 (0.268) | 0.691 |
| gear, lat_cont, lat_sq, hab, dep_sq | 6438.0 | 148.4 | 0.236 (0.165) | 0.306 (0.229) | 0.542 |  | 1269.1 | 9.3 | 0.311 (0.226) | 0.378 (0.268) | 0.689 |
| gear, dep_sq | 9959.0 | 3669.4 | 0.397 (0.269) | 0.334 (0.177) | 0.731 |  | 1278.9 | 19.1 | 0.288 (0.236) | 0.440 (0.341) | 0.729 |
| gear, hab | 9398.7 | 3109.1 | 0.386 (0.283) | 0.359 (0.257) | 0.745 |  | 1278.2 | 18.4 | 0.299 (0.236) | 0.428 (0.305) | 0.727 |
| gear, lat, dep | 6327.5 | 37.9 | 0.246 (0.158) | 0.253 (0.172) | 0.499 |  | 1264.3 | 4.5 | 0.292 (0.239) | 0.343 (0.299) | 0.635 |
| gear, lat, dep_sq | 6374.3 | 84.7 | 0.230 (0.167) | 0.276 (0.187) | 0.506 |  | **1259.8** | **0.0** | 0.282 (0.227) | 0.330 (0.281) | **0.612** |
| gear, lat, hab | 6379.4 | 89.9 | 0.232 (0.173) | 0.283 (0.198) | 0.514 |  | 1261.8 | 2.0 | 0.319 (0.216) | 0.320 (0.282) | 0.639 |
| gear, lat, hab, dep | 6303.8 | 14.3 | 0.240 (0.163) | 0.266 (0.181) | 0.507 |  | 1268.2 | 8.4 | 0.277 (0.243) | 0.387 (0.320) | 0.664 |
| gear, lat, hab, dep_fine | **6289.6** | **0.0** | 0.248 (0.166) | 0.269 (0.181) | 0.517 |  | 1274.9 | 15.1 | 0.251 (0.227) | 0.429 (0.290) | 0.680 |
| gear, lat, hab, dep_sq | 6367.1 | 77.5 | 0.235 (0.173) | 0.283 (0.199) | 0.518 |  | 1263.6 | 3.8 | 0.302 (0.218) | 0.333 (0.300) | 0.635 |
| gear, lat_sq, hab | 6743.0 | 453.5 | 0.242 (0.152) | 0.248 (0.150) | 0.490 |  | 1265.7 | 5.9 | 0.313 (0.239) | 0.354 (0.279) | 0.667 |
| gear, lat_sq, hab, dep | 6517.4 | 227.8 | 0.238 (0.149) | 0.303 (0.199) | 0.541 |  | 1271.3 | 11.6 | 0.316 (0.237) | 0.358 (0.265) | 0.673 |
